# Age biases in a large HIV and sexual behaviour-related internet survey among MSM

**DOI:** 10.1186/1471-2458-13-826

**Published:** 2013-09-10

**Authors:** Ulrich Marcus, Ford Hickson, Peter Weatherburn, Axel J Schmidt

**Affiliations:** 1 Ulrich Marcus, Department of Infectious Disease Epidemiology, Robert Koch-Institute, P.O. Box 650261, Berlin 13302, Germany; 2 Sigma Research, London School of Hygiene and Tropical Medicine, London, UK

**Keywords:** MSM, Internet samples, HIV prevalence, Survey-surveillance discrepancies, Participation bias

## Abstract

**Background:**

Behavioural data from MSM are usually collected in non-representative convenience samples, increasingly on the internet. Epidemiological data from such samples might be useful for comparisons between countries, but are subject to unknown participation biases.

**Methods:**

Self-reported HIV diagnoses from participants of the European MSM Internet Survey (EMIS) living in the Czech Republic, Germany, the Netherlands, Portugal, Sweden and the United Kingdom were compared with surveillance data, for both the overall diagnosed prevalence and for new diagnoses made in 2009. Country level prevalence and new diagnoses rates per 100 MSM were calculated based on an assumed MSM population size of 3% of the adult male population. Survey-surveillance discrepancies (SSD) for survey participation, diagnosed HIV prevalence and new HIV diagnoses were determined as ratios of proportions. Results are calculated and presented by 5-year age groups for MSM aged 15–64.

**Results:**

Surveillance derived estimates of diagnosed HIV prevalence among MSM aged 15–64 ranged from 0.63% in the Czech Republic to 4.93% in the Netherlands. New HIV diagnoses rates ranged between 0.10 per 100 MSM in the Czech Republic and 0.48 per 100 in the Netherlands. Self-reported rates from EMIS were consistently higher, with prevalence ranging from 2.68% in the Czech Republic to 12.72% in the Netherlands, and new HIV diagnoses rates from 0.36 per 100 in Sweden to 1.44 per 100 in the Netherlands. Across age groups, the survey surveillance discrepancies (SSD) for new HIV diagnoses were between 1.93 in UK and 5.95 in the Czech Republic, and for diagnosed prevalence between 1.80 in Germany and 4.26 in the Czech Republic.

Internet samples of MSM were skewed towards younger age groups when compared to an age distribution of the general adult male population. Survey-surveillance discrepancies (SSD) for EMIS participation were inverse u-shaped across the age range. The two HIV-related SSD were u- or j-shaped with higher values for the very young and for older MSM. The highest discrepancies between survey and surveillance data regarding HIV-prevalence were observed in the oldest age group in Sweden and the youngest age group in Portugal.

**Conclusion:**

Internet samples are biased towards a lower median age because younger men are over-represented on MSM dating websites and therefore may be more likely to be recruited into surveys. Men diagnosed with HIV were over-represented in the internet survey, and increasingly so in the older age groups. A similar effect was observed in the age groups younger than 25 years. Self-reported peak prevalence and peak HIV diagnoses rates are often shifted to higher age groups in internet samples compared to surveillance data. Adjustment for age-effects on online accessibility should be considered when linking data from internet surveys with surveillance data.

## Background

Most HIV surveillance systems in Europe provide reasonably good data on number of new diagnoses among men having sex with men (MSM) [1,2]. Data on HIV prevalence are less readily available and less comparable due to different estimation methods and sampling biases. Comparable data on HIV prevalence and incidence among MSM across countries or across different surveys are important to assess population effects of prevention efforts, develop prevention policies and target interventions.

Data on sexual risk behaviours among MSM are increasingly collected by online surveys [3]. Data on recency of HIV testing and self-reported HIV diagnosis are also often collected in these surveys. In 2010, the European MSM Internet Survey (EMIS) demonstrated the feasibility and utility of collecting data from MSM from 38 European countries with the same questionnaire – simultaneously available online in 25 languages – and using the same recruitment methods [4]. However, estimation and comparability of MSM HIV prevalence between countries (and between consecutive surveys) is limited by unknown sizes of MSM populations, differences in household internet access across countries and time [5], Marcus U, Hickson F, Weatherburn P, Schmidt AJ, et al.: Estimating the size of the MSM populations for 38 European countries by calculating the survey-surveillance discrepancies (SSD) between self-reported new HIV diagnoses from the European MSM Internet Survey (EMIS) and surveillance-reported HIV diagnoses among MSM in 2009 (as yet unpublished observations)], and possibly other unknown selection effects. For example, in EMIS, although a broad range of websites were used for recruitment, participation rates varied substantially even between countries with similar household internet access. Substantial differences between national samples were also observed regarding median age, even between countries with very similar socio-cultural, political and economic background and similar histories and starting points of the HIV epidemic among MSM – like e.g. Germany and the Netherlands, two neighbouring countries in the centre of Europe [4].

Since the internet sites most commonly used for survey recruitment are dating and ‘cruising’ sites, it can be expected that samples recruited this way over-represent more sexually active MSM [6]. In addition to men using these sites not being representative of all MSM (and likely not being used equally across the age range), an unknown participation bias will also be in operation. For both these reasons the age distribution of samples recruited on these sites may differ from the actual age distribution of MSM. Because partner numbers and sexual activity decline with age [7], older men in particular may be expected to be under-represented.

In previous analyses we have looked into discrepancies between self-reported EMIS data and surveillance data on the prevalence and incidence of diagnosed HIV among MSM by comparing on a country level [5], Marcus U, et al.: Estimating the size of the MSM populations for 38 European countries by calculating the survey-surveillance discrepancies (SSD) between self-reported new HIV diagnoses from the European MSM Internet Survey (EMIS) and surveillance-reported HIV diagnoses among MSM in 2009 (as yet unpublished observations)]. In cross-country comparisons household access to the internet was a major determinant of participation rates and survey surveillance discrepancies (SSD). This study seeks to measure the differences in rates of age-specific survey participation, new HIV diagnoses, and HIV prevalence between MSM who participate in internet surveys and the general MSM population. By adjusting for these differences, cross-country comparisons in findings from internet surveys can be made with greater validity.

## Methods

### Selection of countries

Among the 38 countries with sample sizes larger than 100 respondents in EMIS we selected the following countries (in alphabetical order): the Czech Republic (Central East Europe); Germany (Central West Europe); the Netherlands (West Europe); Portugal (South West Europe); Sweden (North West Europe); the United Kingdom (West Europe). Countries were selected to represent a variety of European sub-regions and varying EMIS participation rates. Further requirements were a sufficient size of the EMIS sample, and availability of relatively reliable HIV surveillance data regarding MSM.

### HIV surveillance data and population statistics

We set the lower and upper age limits of both the EMIS sample and the surveillance data to be 15 years and 65 years. For the Czech Republic, as the only country from an eastern European sub-region, we also analysed the data for the narrower age range of 15 to 49 years, because the HIV epidemic among MSM in the eastern parts of Europe started about 10–15 years later than in the western parts, leading to a different age distribution of HIV infections in the MSM population.

Data on new HIV diagnoses in 2009 were taken from national infectious disease surveillance systems. Cases with unknown risk factors for HIV acquisition were proportionately redistributed based on known cases.

For surveillance measures of HIV prevalence we used diagnosed infections only in order to compare with self-reported prevalence, which is also a diagnosed prevalence. Estimates of the number of MSM with diagnosed HIV were based on a Multi-parameter Evidence Synthesis (MPES) approach for the Netherlands [8], on a back-calculation model for Germany and the United Kingdom [9,10], on the proportion of MSM among people infected with HIV and in clinical care for Sweden (InfCareHIV database), the Czech Republic and Portugal (similar databases on people infected with HIV in clinical care; personal communications).

The total size of the adult male population aged 15 to 64 years (or 15 to 49 years for Czech Republic) was taken from national population statistics [11-16]. The relative size of the MSM population was estimated to be 3% in all age groups except males aged less than 20. This estimate is consistent with the upper limit of the confidence interval of men reporting male sexual partners in the previous 12 months in repeated telephone surveys in representative samples of the general population in Germany conducted by the German Federal Agency of Health Promotion (BZgA) [17] and with published results from a large British national probability survey conducted in 2000 [18]. In Portugal, an unpublished population based study from 2007 also found a proportion of 3% men reporting sex with men in the adult male population in the previous year [H. Barros, personal communication], while a Czech study from 2008 found a proportion of 1.7% reporting repeated sexual contacts with other men [19]. For males below the age of 20 the proportion of MSM was estimated based on the proportion of EMIS respondents in the national samples reporting their first sexual experience with another man before the age of 20 [4]. For these men the proportion was estimated to be 2.1% (70% of 3%), since sex with a male partner before the age of 20 was reported by 70% of the EMIS respondents in the six countries.

Due to the lower social acceptance of homosexuality in the eastern parts of Europe we varied the proportion of MSM in the male adult population for the Czech Republic to include 2%, so that for the Czech Republic we present calculated values for a MSM population covering a range from 2% of the adult population 15–49 years up to 3% of the 15–64 year old adult male population.

### Self-reported data on prevalent and newly diagnosed HIV infections in 2009

EMIS was a large scale pan-European internet survey conducted in 2010. The methodology has been described in more detail elsewhere [20]. In brief, a network of five primary and 77 secondary partners working in MSM sexual health across academia, public health and community organizations in 38 European countries developed a collaborative English language survey. The survey was translated into 24 other languages, and promoted through gay dating websites and through gay community organizations. EMIS was approved by the Research Ethics Committee of the University of Portsmouth, United Kingdom (REC application number 08/09:21).

Among other questions, survey participants were asked about the year of birth, their age when they first had sex with a man, the result of their last HIV test, and the recency of that test if it was negative, or the year of first diagnosis if it was positive.

### Comparison of surveillance and self-reported data

Assuming a stable proportion of MSM in the adult male population once a homosexual debut has occurred, the age distribution of the EMIS samples was compared to the age distribution of the general population, taking into account a reduced proportion of MSM below the age of 20.

Self-reported prevalence rates (per hundred EMIS respondents, regardless of having been tested for HIV) and new diagnoses rates in 2009 per 100 EMIS respondents were compared with the prevalence and incidence of diagnosed infection calculated from surveillance data and population estimates. The denominators for the rate of newly diagnosed HIV in 2009 were the total national EMIS sample for self-reports and the estimated total MSM population for surveillance data.

Comparisons were made by calculating a ratio of the proportions with EMIS data in the numerator and population/surveillance data in the denominator. This ratio of proportions we call the Survey-Surveillance Discrepancy (SSD) [5,21]. We calculated the SSD for EMIS participation (proportional distribution of EMIS participants by age groups/proportional age distribution of the total male population), the SSD for prevalence (self-reported prevalence rate in EMIS by age group/estimated diagnosed prevalence in the MSM population), and the SSD for new HIV diagnoses (self-reported HIV diagnoses in 2009 per 100 EMIS respondents/reported new HIV cases per 100 MSM based on surveillance data). Particularly for age-group data on new HIV diagnoses in 2009 the numbers can get quite small and precision of SSD calculation is thus affected by chance effects. Therefore for comparisons of countries we used the best fitting 2^nd^ order polynomic trendlines imposed on the data curves. Despite the problem of low numbers we looked at new diagnoses data, because these data are more readily available from national surveillance systems than prevalence data/estimates.

## Results

In Table 1 we present, for each of the six countries analysed:

•The number of men aged 15 to 64;

•The estimated size of the MSM population;

•The rate of newly diagnosed HIV per 100 MSM based on surveillance reports;

•The prevalence of diagnosed HIV infections per hundred MSM based on surveillance reports;

•The national EMIS sample sizes;

•Self-reported HIV diagnoses in 2009 per 100 EMIS respondents;

•Self-reported diagnosed HIV infections per hundred EMIS respondents;

•The EMIS participation rates per 10,000 adults (15–64 years);

•The response rates to individualized instant messages sent to men on the two most productive recruitment websites inviting members to participate in EMIS;

•The median age of the national EMIS samples;

•The proportion of households with broadband internet access in 2009.

**Table 1:** Population data, surveillance data and EMIS derived data to characterize and compare the six national MSM samples

	**Population**			**Surveillance**		**EMIS**					
Country	Adult male population (15–64 years)	Estimated MSM population (15–64)	Proportion of households with broadband Internet access in 2009 (%)	HIV diagnoses in MSM per 100 MSM in 2009	Estimated proportion of MSM diagnosed with HIV by end of 2009 (%)	EMIS sample size	HIV diagnoses in 2009 per 100 EMIS respondents	Proportion of EMIS respondents diagnosed with HIV by end of 2009 (%)	EMIS participation rate per 10,000 adults	Response rates to individualized invitation instant messages (%)	Median age of EMIS participants (years)
Czech Republic (1)	3654797	109644	46%	0.10	0.63%	2392	0.59	2.68%	2.30	8.70%	27
Czech Republic (2)	2561675	51234		0.21	1.34%	2284	0.61	2.71%	4.25		
Germany	27299462	793498	65%	0.32	4.29%	53653	0.74	7.66%	6.60	11.00%	33
Netherlands	5589000	163035	77%	0.48	4.93%	3696	1.44	12.72%	2.30	7.90%	40
Portugal*	3524528	103153	46%	0.33	3.58%	5158	0.78	7.75%	4.90	11.80%	30
Sweden	3098248	89999	69%	0.15	1.67%	3058	0.36	4.81%	3.40	8.00%	35
United Kingdom	20545700	598256	71%	0.46	4.51%	17362	0.89	10.44%	3.00	4.90%	36

(1) population 15–64 years, 3% MSM; (2) population 15–49 years, 2% MSM; * the estimate of MSM in care from Portugal includes an unknown number of unreported deaths. Thus this estimate may be slightly too high. *MSM* Men having sex with men, *EMIS* European Men-Who-Have-Sex-With-Men Internet Survey.

Diagnosed HIV prevalence among MSM aged 15–64 was estimated to be between 0.63% (3% MSM, 15–64) and 1.34% (2% MSM, 15–49) in the Czech Republic and 4.93% in the Netherlands by the end of 2009. The rate of new diagnoses with HIV in 2009 ranged between 0.10 per 100 MSM (3%, 15–64) in the Czech Republic and 0.48 in the Netherlands. Contrastingly, self-reported prevalence in EMIS respondents ranged between 2.68% in the Czech Republic and 12.72% in the Netherlands. The rate of respondents reporting being newly diagnosed with HIV in 2009 varied between 0.36 per 100 in Sweden and 1.44 per 100 in the Netherlands (Table 1; see also Additional file 1).

**Figure 1:**
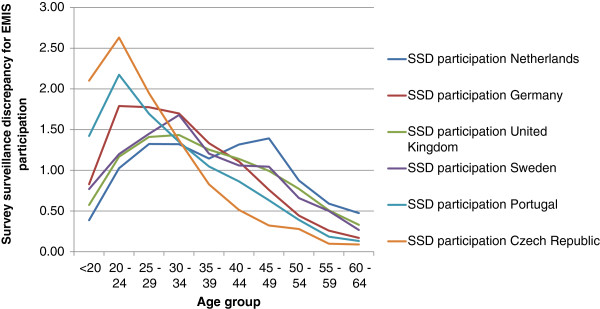
Survey-surveillance discrepancy curves for EMIS participation (=proportion of EMIS respondents per age group/ proportion of male adults per age group) for six EMIS countries: Czech Republic (CZ), Germany (DE), The Netherlands (NL), Portugal (PT), Sweden (SE), United Kingdom (UK).

Figure 1 shows the age-group related SSD curves for EMIS participation in the six countries. Values above 1 mean that the respective age group was relatively over-represented among EMIS respondents. The Czech and the Portuguese samples had the lowest median age; the Dutch sample the highest. In all samples, men younger than 20 were less well represented than men aged 20–24 years, even if we include only 70% of the known population of 15-19 year olds, because of our lower estimate of homosexual activity in the youngest group. In the Czech Republic and Portugal men younger than 20 were still over-represented compared with the general population. In Sweden, MSM up to the age of 30 were less well reached by the recruitment websites than in the other countries. Men aged above 40 were reached particularly badly in the Czech Republic and particularly well in the Netherlands.

**Figure 2:**
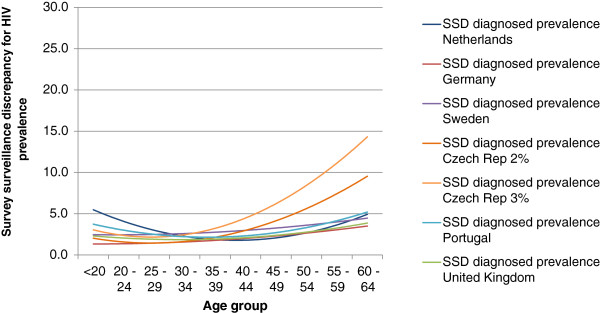
**2**^**nd **^**order polynomic trendline curves for survey-surveillance discrepancies between self-reported HIV prevalence in EMIS and diagnosed HIV prevalence based on surveillance data (self-reported HIV prevalence per 5-year age group in EMIS/ diagnosed prevalence per age group in the MSM population).**

Figure 2 shows the age group related 2^nd^ order polynomic trendline curves for the SSD between self-reported HIV prevalence in EMIS and diagnosed HIV prevalence based on surveillance data. The curves were U or J-shaped with higher SSD values for age groups which were relatively under-represented. The curves for the countries in western parts of Europe were very similar, while the curves for the Czech Republic (regardless of which MSM population estimate was used) showed larger differences for the higher age groups.

**Figure 3:**
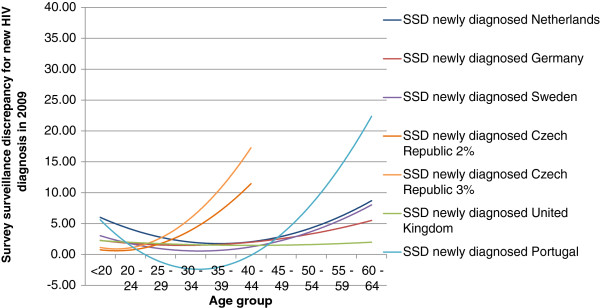
**2**^**nd **^**order polynomic trendline curves for survey-surveillance discrepancies between self-reported new HIV diagnosis in 2009 in EMIS and surveillance data for 2009 (=number of HIV infections diagnosed in 2009 per 100 EMIS participants/ number of newly diagnosed HIV infections in 2009 per 100 MSM by 5-year age group).**

**Figure 4:**
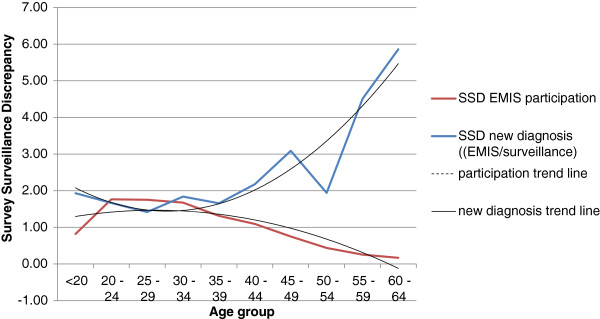
Relationship between the SSD for EMIS participation and the SSD for new HIV diagnoses, taking Germany as example.

Figure 3 shows age group related 2^nd^ order polynomic trendline curves for survey-surveillance discrepancies between self-reported new HIV diagnoses in 2009 in EMIS and surveillance data for 2009. Here again, the curves of all countries in western parts of Europe were U-shaped, but SSD for men below 20 could not be calculated for some countries (NL, SE) because the EMIS samples did not contain men from this age group diagnosed with HIV in 2009. The curve for the Czech Republic was J-shaped and shorter, because no infections were diagnosed in EMIS participants younger than 20 and older than 45 in 2009. Compared with the SSD for prevalence, the SSD for new diagnoses showed higher discrepancies for Portugal: However, this may be mainly due to low numbers of newly diagnosed HIV infections in the EMIS sample particularly in higher age groups and resulting chance effects on SSD for higher age groups. Figure 4 shows the relationship between the SSD for EMIS participation and the SSD for new HIV diagnoses taking Germany as an example: for less well represented age groups, discrepancies between measured and self-reported new diagnoses increase, suggesting that in these age groups men with HIV were increasingly disproportionately likely to participate in EMIS.

Table 2 presents the overall (not age group related) values for the SSD for prevalence and new diagnoses for the six countries. SSDs both for prevalence and new HIV diagnoses in the countries in western parts of Europe are remarkably similar, with the Netherlands ranging at the upper end of the distribution, but also having the lowest participation rate of the western countries despite a high household internet access rate (see Table 1). The SSDs for the Czech Republic are in the same range as the western European countries when calculating with a MSM population size of 2% of the 15–49 year old male population. The values become twice as high as in the western parts of Europe if using a population size of 3% of the 15–64 year old male population.

**Table 2:** Overall SSD for prevalence and for new HIV diagnoses

**Country**	**SSD for diagnosed HIV prevalence**	**SSD for newly diagnosed HIV**
Czech Republic (1)	4.26	5.95
Czech Republic (2)	2.37	2.89
Germany	1.80	2.32
Netherlands	2.58	3.04
Portugal	2.16	2.32
Sweden	2.89	2.39
United Kingdom	2.31	1.93

(1) population 15–64 years, 3% MSM; (2) population 15–49 years, 2% MSM; *SSD* survey-surveillance discrepancy.

## Discussion

The proportional age distributions of large internet samples of MSM recruited mainly via gay social media (dating websites) were skewed towards younger age groups – except for males younger than 20 years - when compared to the age distribution of adult males. The proportion of men accessible on Internet dating sites declines once they get older than 45 years.

Participants of the internet survey had a higher risk for lifetime and recent HIV diagnosis than a hypothetical random sample of MSM, provided the proportion of MSM among adult males in the countries is closer to 3% than to 1.0% or 1.5%. Particularly among men older than 45 years it was observed that those who are still accessible and participate in an HIV-related survey are increasingly biased towards higher lifetime and recent risks for HIV. This biased age representation in internet samples results in a peak of self-reported HIV prevalence and new HIV diagnoses in an older age group than measured by surveillance data. From the six countries analysed here, this happened in four countries when we considered HIV prevalence, and all six countries when we considered new HIV diagnoses.

In most age groups and most countries – except the relatively overrepresented young age groups in the Czech Republic and Portugal – both HIV-related SSDs were higher than 1.0., and in four of the six countries the SSD for new HIV diagnoses was slightly higher than the SSD for (diagnosed) prevalence, suggesting one or both of the following:

•HIV diagnosis, including a recent HIV diagnosis is a strong motivation to participate in a HIV-related survey;

•MSM accessible on internet dating sites have higher odds of being diagnosed with HIV than a random MSM sample.

A fourfold higher risk for an STI diagnosis among MSM participating in a community based internet study compared with MSM from a population based probability sample was also reported from a UK study [22].

A variety of different factors could be responsible for reduced accessibility of younger (15–19) and older (50+) MSM on internet dating websites. While we have supporting data on sexual debut to explain the paucity of younger men, we have no data that could explain reduced accessibility of older age groups. More research will be required to elucidate the reasons for this. We speculate that lower internet literacy, less sexual partner change, and increasing proportions living in settled relationships may be among these factors.

**Figure 5:**
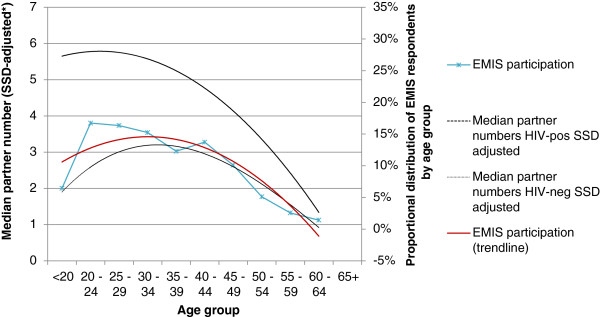
Self-reported median number of non-steady sexual partners per age group in the German EMIS sample (SSD-adjusted*), stratified by self-reported HIV status: correlation with EMIS age distribution. * Reported median partner numbers were divided by the age group-related SSD factor.

If we take into consideration that older survey participants were more likely to be at higher risk for HIV, self-reported partner numbers for older survey participants probably over-estimate partner numbers of older MSM in the population. When looking at median self-reported partner numbers among EMIS respondents (see Figure 5), and tentatively adjusting them for the biased age representation in internet samples, age distribution of median partner numbers closely correlates with age distribution of EMIS respondents. This may suggest that internet samples do represent very well the part of the MSM population which is at risk for HIV and STIs through frequent partner change.

Previous research in Germany has shown that regarding geographical distribution of newly diagnosed cases of HIV, large internet samples can be representative of MSM populations [23]. The discrepancy between skewed age distribution of internet samples and good representation of new HIV diagnoses suggests that internet samples may very adequately represent the sexually active MSM population most at risk for HIV.

**Figure 6:**
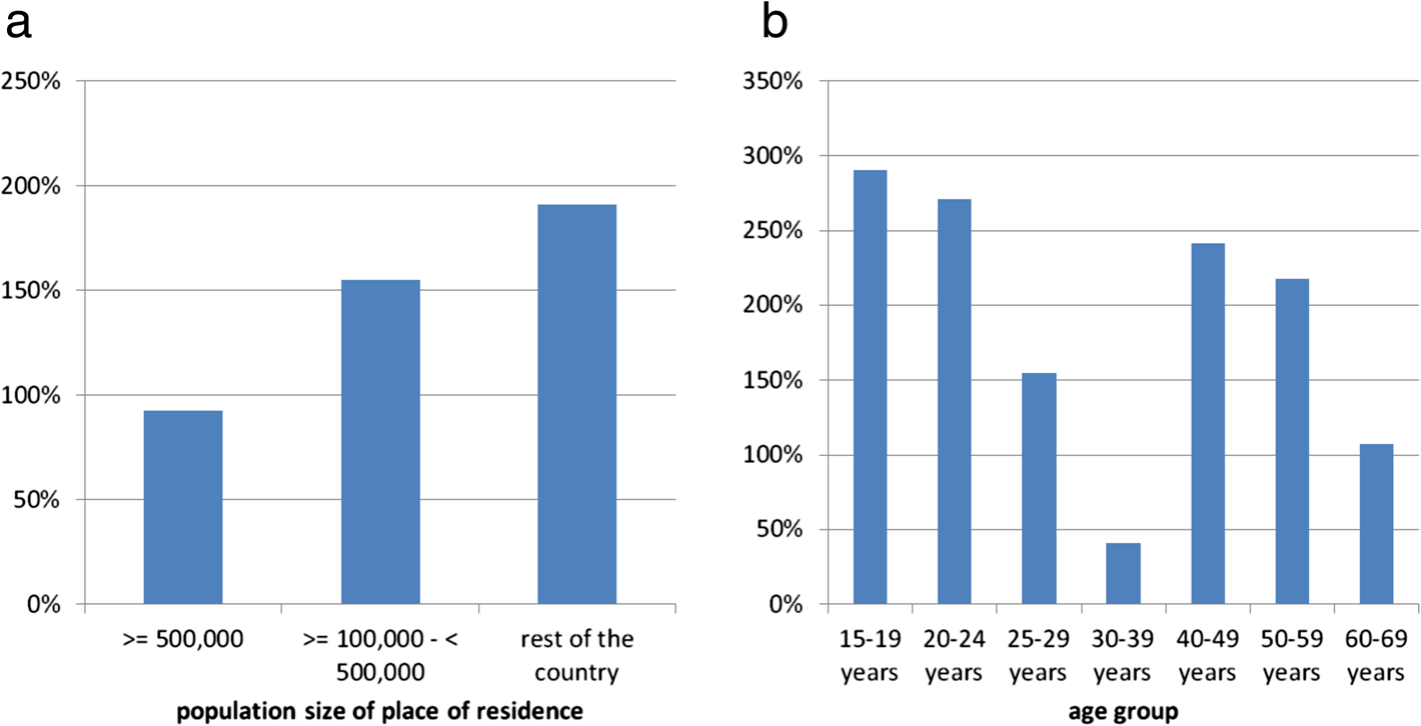
**Changes of newly diagnosed HIV infections among MSM in Germany between 2001–2003 and 2010–2012.** Figure 6**a**: Proportional increase by population size of the place of residency Figure 6**b**: Proportional increase by age group.

HIV-related SSDs and MSM population estimates are intrinsically linked. The example of the Czech Republic demonstrates that when we increase the estimate of the percent of the overall population that is MSM, the SSD value also increase, if we decrease this estimate, so does the SSD value. The same applies to higher SSD values for older and younger MSM age groups: If we assume that the MSM population in these age groups is smaller than 3% respectively 2.1% of the male adult population, differences in SSD values between age groups would become smaller. Ultimately the interpretation of the relation between these two factors depends on the conceptualization of the issue or the definition of the MSM population: do internet surveys recruit MSM with higher HIV risk who self-select from a broader MSM population or do they recruit MSM representative of an MSM population which is defined by being susceptible for HIV due to a high level of connectedness? Connectedness in this sense has two dimensions: to be able to identify and contact each other, which is strongly facilitated by MSM-specific websites, and to be able to actually meet each other, which requires either a high population density or high mobility. This would imply that the size of this epidemiologically connected population is not stable, but increased in recent years due to wider availability of internet access, and will further increase in countries with low levels of household internet access. That such an expansion of the MSM population may have occurred is suggested by epidemiological data from Germany: between the period 2001–2003 and 2010–2012 (a time when internet access in Germany increased substantially) new HIV diagnoses among MSM disproportionately increased in the younger and older age groups and in men living outside of the large cities, i.e. in men traditionally less well connected to the “gay scene” (see Figures 6a and 6b). From this perspective, low SSD values would signal a highly connected population, high SSD values a less well connected population. However, this remains speculation and requires further research.

It would be interesting to repeat a survey like EMIS and to look at changes particularly in countries with increasing household access to Internet. Also, it would have been interesting to analyse SSDs for more countries in eastern parts of Europe. However, for many smaller countries the EMIS samples were too small for this kind of analysis (numbers of men with HIV in age groups become too small), and particularly for the larger countries (Russia, Ukraine, Poland, Romania) surveillance data with regard to the size of the MSM transmission group are unreliable. One way to circumvent the issue of smaller sample sizes would be to use cumulated diagnoses numbers over several years for SSD calculations.

While the above interpretation of SSDs explains well the differences observed between western and eastern Europe and between a less densely populated country like Sweden and densely populated countries like Germany and England, it would not explain the SSD differences between the Netherlands and countries in the other western parts of Europe. The higher SSDs in the Netherlands, together with the lower participation rates, may indicate real differences in selection biases of survey participants. A possible reason for a different self-selection bias could be the high frequency of national MSM Internet surveys in the Netherlands (yearly), and the launch of a national survey for MSM shortly before the launch of the European survey (EMIS) in 2010 which may have resulted in survey fatigue effects in the target population.

Our paper on the limitations of data from internet convenience samples itself has some limitations, not least because we are using data from an internet convenience sample, which we know are not representative. There are also representation limitations to our other sources of data, censuses and HIV surveillance, most crucially that the prevalence estimates from surveillance data from the six countries are based on different methods. While the estimates for Germany, the Netherlands and UK on the one side and for Czech Republic, Portugal and Sweden on the other side may be largely comparable, there may be some differences between these two groups of countries. In the datasets for MSM in clinical care, men with an early HIV diagnosis not meeting the thresholds for starting antiretroviral treatment may be slightly under-represented. This may disproportionally affect younger age groups. However, since SSD values particularly for the younger age groups are already quite low in these countries, it seems unlikely that diagnosed HIV prevalence is substantially underestimated. Last but not least, our knowledge of the proportion of the male population who are homosexually active is weak, especially across the age range.

## Conclusions

Increasing underrepresentation of older men on internet dating sites was associated with an increasing bias towards men with diagnosed HIV. Through this phenomenon self-reported peak prevalence and new HIV diagnoses rates may be shifted to higher age groups in internet samples than in the population.

For many comparisons between national EMIS samples it may be unnecessary to take biased age representations into account, because it is unlikely to change the ranking between the countries. However, when survey data are linked to surveillance data, survey data are used to interpret surveillance data or survey data are used to make statements on the MSM population it may be advisable to take survey surveillance discrepancies into account.

If reasonably reliable prevalence estimates or data on new HIV diagnoses by age groups are available, a survey surveillance discrepancy factor for internet based or other convenience samples can be calculated for adjusting survey data for the total population, if necessary.

## Competing interests

The authors declare that they have no competing interests.

## Authors’ contributions

UM, FH, PW, and AJS participated in the design of the survey tool for the internet survey. UM instigated the Internet survey, led the study design, and drafted the manuscript. FH participated in the study design, constructed the online survey and contributed to the manuscript; PW participated in the study design, co-ordinated the survey promotion and contributed to the manuscript; AJS participated in the study design, coordinated the study and the EMIS Network, and contributed to the manuscript. All authors approved the final manuscript.

## Funding

The EMIS project was funded by EAHC - Executive Agency for Health and Consumers, EU Health Programme 2008–2013 (funding period: 14.3.2009 - 13.9.2011).

CEEISCat - Centre d’Estudis Epidemiològics sobre les ITS/HIV/SIDA de Catalunya (2009–2012); Terrence Higgins Trust (CHAPS) for Department of Health for England (2009–2012); Maastricht University (2009–2012); Regione del Veneto (2009–2012); Robert Koch Institute (2009–2012); BZgA (Bundeszentrale für gesundheitliche Aufklärung, Köln: 2010–2011); German Ministry of Health (2010); Finnish Ministry of Health (2010); Norwegian Institute of Public Health (2010–2011); Swedish Board of Health and Welfare (2010–2011).

## The EMIS Network consists of

Associated Researchers: Rigmor A. Berg (Norwegian Knowledge Centre for the Health Services, Oslo); Michele Breveglieri (Regione del Veneto, Verona); Laia Ferrer (CEEISCat, Barcelona); Percy Fernández-Davila (CEEISCat, Barcelona); Cinta Folch (CEEISCat, Barcelona); Martina Furegato (Regione del Veneto, Verona); Ford Hickson (Sigma Research, London); Harm J. Hospers (University College Maastricht); Ulrich Marcus (Robert Koch Institute, Berlin); David Reid (Sigma Research, London); Axel J. Schmidt (Robert Koch Institute, Berlin); Todd Sekuler (Robert Koch Institute, Berlin); Peter Weatherburn (Sigma Research, London).

National collaborating partners of the EMIS Network: Aids-Hilfe Wien (Austria); Facultés Universitaires Saint-Louis, Institute of Tropical Medicine, Ex Aequo, Sensoa, Arc-en-ciel (Belgium); Vstrecha (Belarus); National Centre of Infectious and Parasitic Diseases, Queer Bulgaria Foundation (Bulgaria); Charles University, Institute of Sexology (Czech Republic); Research Unit in Behaviour & Social Issues (Cyprus); University of Zagreb, Faculty of Humanities and Social Sciences (Croatia); Statens Serum Institut, Department of Epidemiology, stopaids (Denmark); National Institute for Health Development (Estonia); University of Tampere, Department of Nursing Science, Finnish AIDS council (Finland); Institut de veille sanitaire (InVS), AIDeS, Act UP Paris, Sida Info Service, Le kiosque, The Warning (France); Berlin Social Science Research Center (WZB), Deutsche AIDS-Hilfe (DAH), Federal Centre for Health Education, Cologne (BZgA) (Germany); Positive Voice (Greece); Hungarian Civil Liberties Union, Háttér (Hungary); Gay Men’s Health Service, Health Services Executive (Ireland); University of Bologna, Italian Lesbian and Gay Association (Arcigay), Instituto Superiore di Sanità (National AIDS Unit) (Italy); The Infectiology Center of Latvia, Mozaika (Latvia); Center for Communicable Diseases and AIDS (Lithuania); GenderDoc-M (Moldova); schorer (Netherlands); Norwegian Knowledge Centre for the Health Services, The Norwegian Institute of Public Health (Norway); National AIDS Centre, Lamda Warszawa (Poland); GAT Portugal, University of Porto, Medical School, Inst. of Hygiene and Tropical Med. (Portugal); PSI Romania (Romania); PSI Russia (Russia); Safe Pulse of Youth (Serbia); OZ Odyseus (Slovakia); National Institute of Public Health, SKUC-Magnus, Legebitra, DIH (Slovenia); National Centre of Epidemiology, stopsida, Ministerio de Sanidad, Política Social e Igualdad (Spain); Malmö University, Health and Society, RFSL, National Board of Health and Welfare (Sweden); Institut universitaire de médecine sociale et preventive, Aids-Hilfe Schweiz (Switzerland); Turkish Public Health Association, Siyah Pembe Üçgen İzmir, KAOS-GL, Istanbul-LGBTT (Turkey); Gay Alliance, Nash Mir, LiGA, Nikolaev (Ukraine); City University London, Department for Public Health, Terrence Higgins Trust and the CHAPS partners including GMFA, The Eddystone Trust, Healthy Gay Life, The Lesbian and Gay Foundation, The Metro Centre London, NAM, Trade Sexual Health, Yorkshire, MESMAC (United Kingdom).

European Collaborating Partners: International Gay and Lesbian Organization (ILGA); European AIDS Treatment Group (EATG); PlanetRomeo.com; Manhunt and Manhunt Cares.

## Pre-publication history

The pre-publication history for this paper can be accessed here:

http://www.biomedcentral.com/1471-2458/13/826/prepub

## Supplementary Material

Additional file 1 Self-reported and reported HIV prevalence in MSM by 5-years age groups in, Germany, United Kingdom, the Netherlands, Sweden, Portugal, and the Czech Republic. Click here for file
