# Supplementary material for: Age biases in a large HIV and sexual behaviour-related internet survey among MSM
Source: BMC Public Health. 2013 Sep 10;13:826. doi: 10.1186/1471-2458-13-826 (PMC3847490; doi:10.1186/1471-2458-13-826)
Supplement: Additional file 1 — Self-reported and reported HIV prevalence in MSM by 5-years age groups in, Germany, United Kingdom, the Netherlands, Sweden, Portugal, and the Czech Republic. [file 1471-2458-13-826-S1.pdf]

Additional figures:
Self-reported and reported HIV prevalence in MSM by 5-year age groups in Germany, United Kingdom, the Netherlands, Sweden, Portugal, and the Czech Republic.

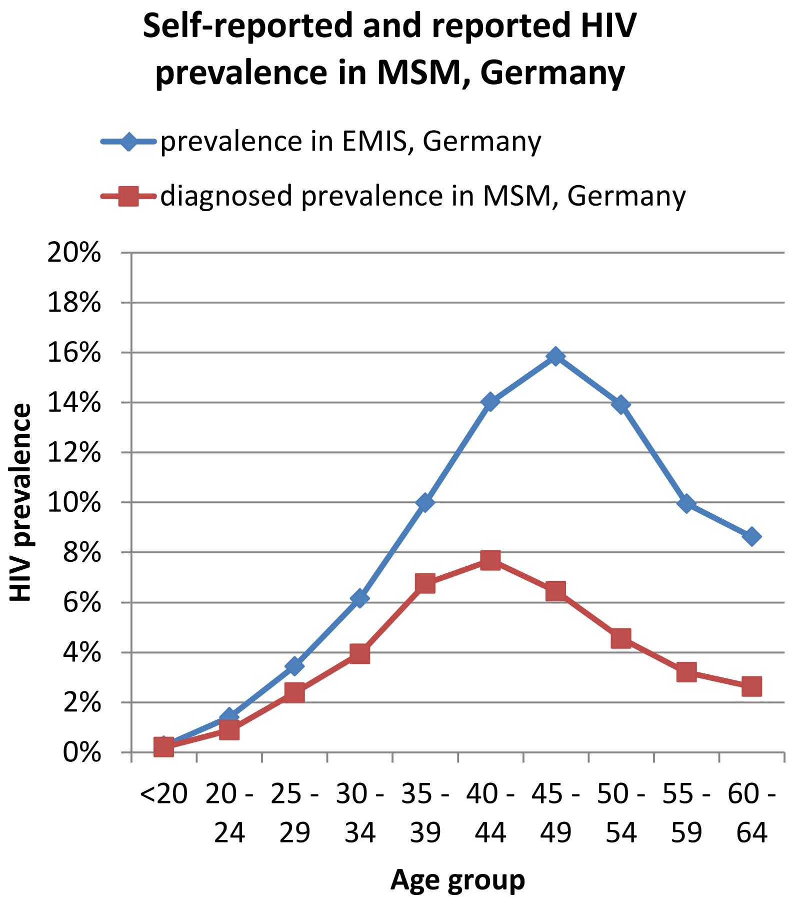

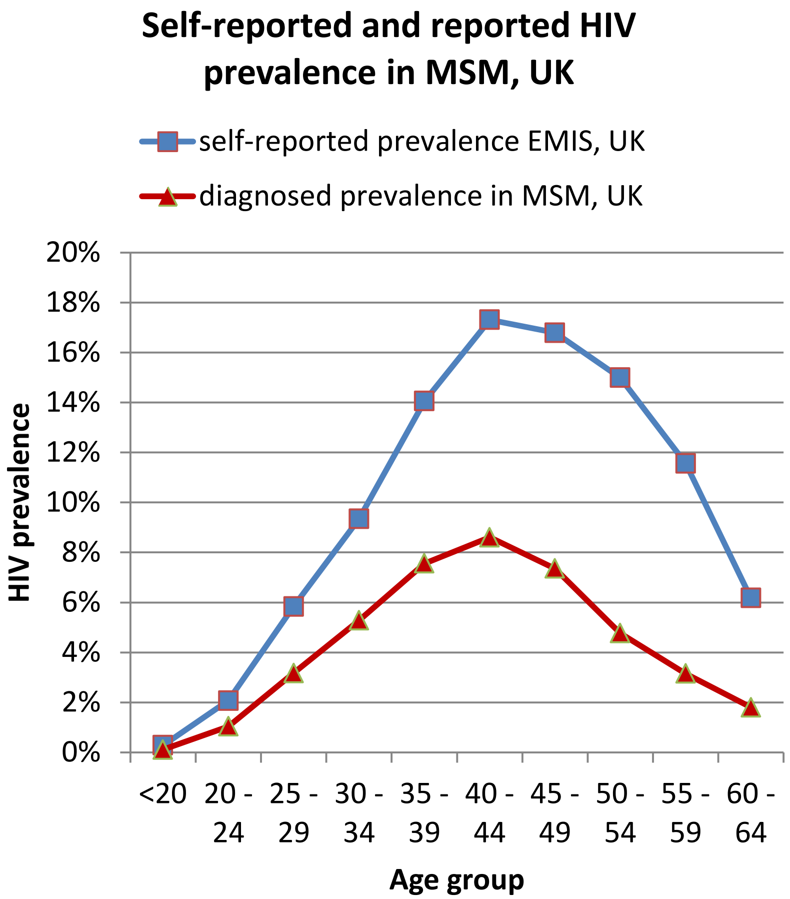

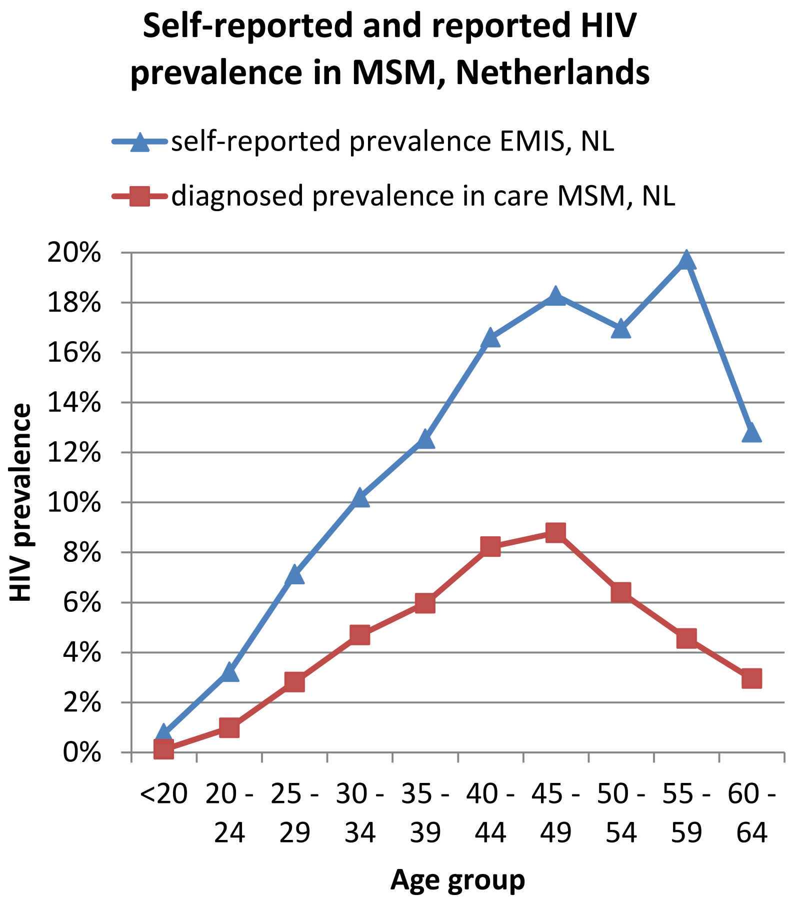
Self-reported and reported HIV prevalence in MSM, Netherlands
self-reported prevalence EMIS, NL
diagnosed prevalence in care MSM, NL
HIV prevalence
20%
18%
16%
14%
12%
10%
8%
6%
4%
2%
0%
<20
20 - 24
25 - 29
30 - 34
35 - 39
40 - 44
45 - 49
50 - 54
55 - 59
60 - 64
Age group

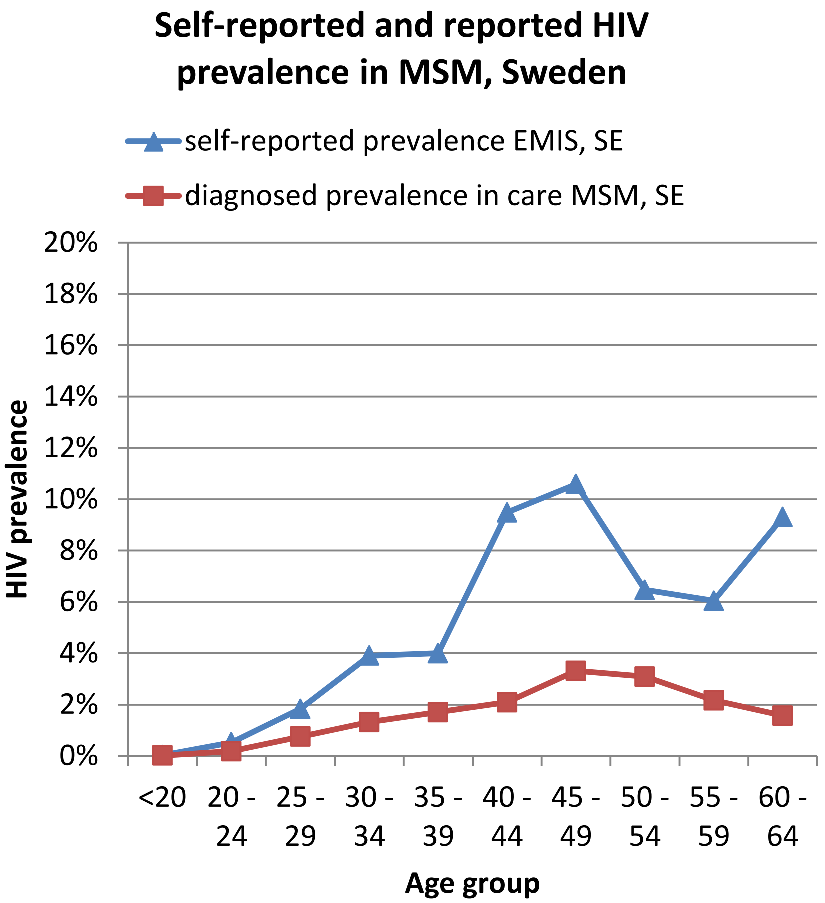
Self-reported and reported HIV prevalence in MSM, Sweden
self-reported prevalence EMIS, SE
diagnosed prevalence in care MSM, SE
HIV prevalence
20%
18%
16%
14%
12%
10%
8%
6%
4%
2%
0%
<20
20 - 24
25 - 29
30 - 34
35 - 39
40 - 44
45 - 49
50 - 54
55 - 59
60 - 64
Age group

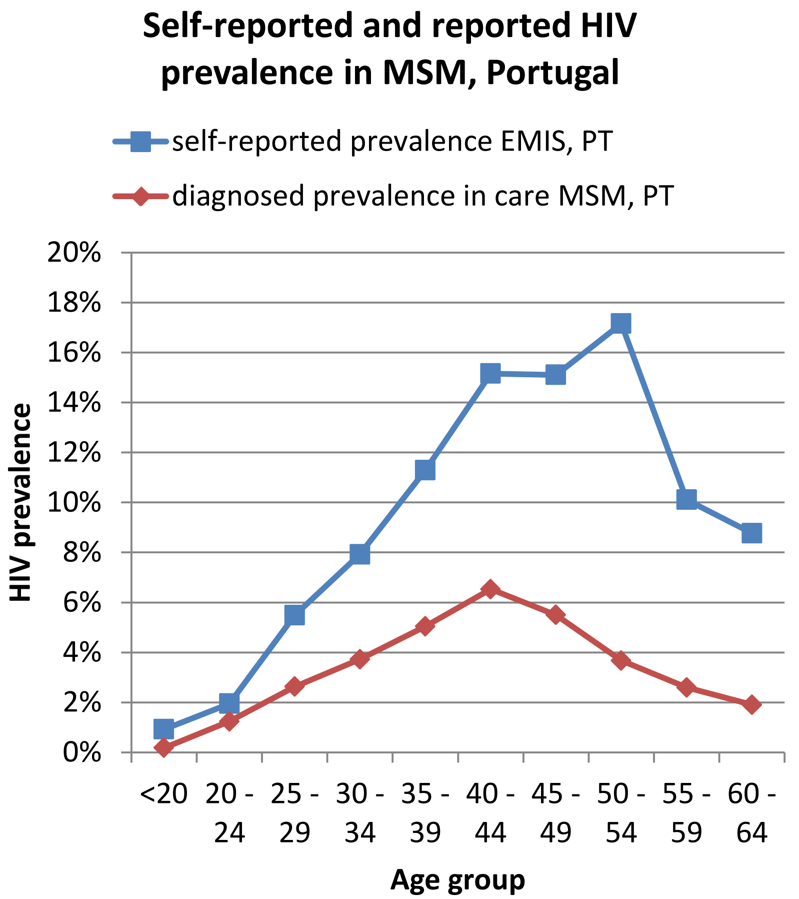
Self-reported and reported HIV prevalence in MSM, Portugal
self-reported prevalence EMIS, PT
diagnosed prevalence in care MSM, PT
20%
18%
16%
14%
12%
10%
8%
6%
4%
2%
0%
HIV prevalence
<20
20 - 24
25 - 29
30 - 34
35 - 39
40 - 44
45 - 49
50 - 54
55 - 59
60 - 64
Age group

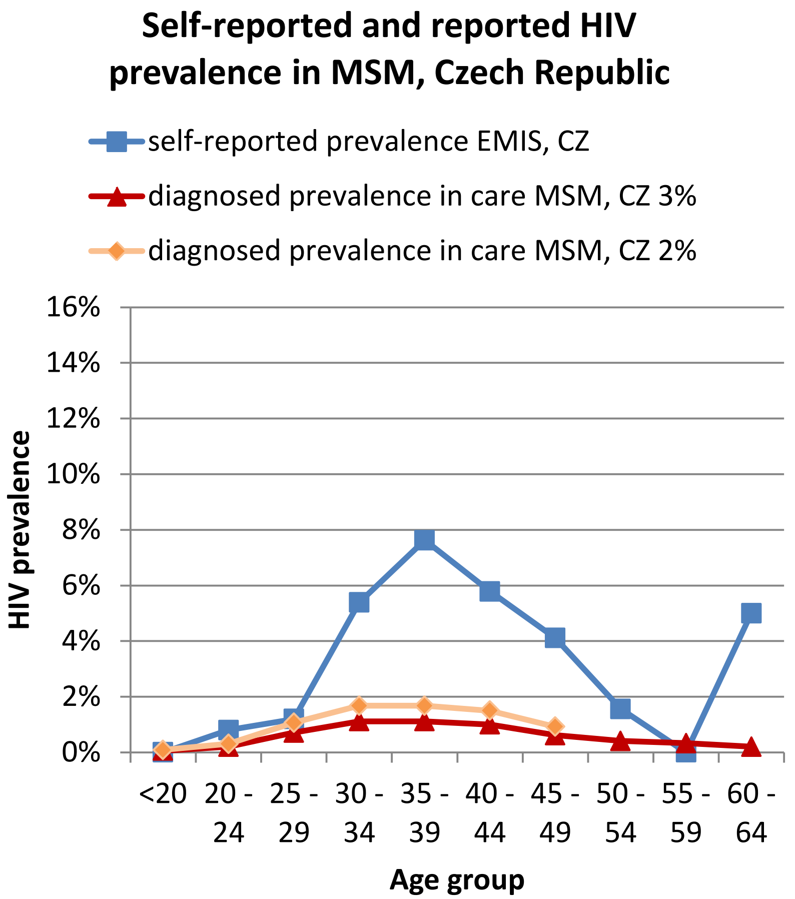
Self-reported and reported HIV prevalence in MSM, Czech Republic
self-reported prevalence EMIS, CZ
diagnosed prevalence in care MSM, CZ 3%
diagnosed prevalence in care MSM, CZ 2%
16%
14%
12%
10%
8%
6%
4%
2%
0%
HIV prevalence
<20
20 - 24
25 - 29
30 - 34
35 - 39
40 - 44
45 - 49
50 - 54
55 - 59
60 - 64
Age group
